# *Klebsiella pneumoniae* bacteremia and renosplenic abscesses without intestinal symptoms as the initial manifestations of non-steroidal anti-inflammatory drug-induced colitis: a rare case report

**DOI:** 10.1186/1471-230X-13-139

**Published:** 2013-09-22

**Authors:** Hung-Ling Huang, Po-Liang Lu, Chun-Yu Lin, Yen-Hsu Chen, Chao-Hung Kuo, Wei-Ru Lin

**Affiliations:** 1 Division of Infectious Diseases, Department of Internal Medicine, Kaohsiung Medical University Hospital, Kaohsiung Medical University, 100, Tzyou 1st Road, Kaohsiung 807, Taiwan; 2 Division of Gastroenterology, Department of Internal Medicine, Kaohsiung Medical University Hospital, Kaohsiung Medical University, Kaohsiung, Taiwan; 3 Department of Internal Medicine, Kaohsiung Medical University Hospital, Kaohsiung Medical University, Kaohsiung, Taiwan; 4 School of Medicine, College of Medicine, Kaohsiung Medical University, Kaohsiung, Taiwan; 5 Graduate Institute of Medicine, College of Medicine, Kaohsiung Medical University, Kaohsiung, Taiwan

**Keywords:** Non-steroidal anti-inflammatory drugs, Colitis, Bacteremia, *Klebsiella pneumoniae*, Renosplenic abscesses

## Abstract

**Background:**

Non-steroidal anti-inflammatory drugs (NSAIDs), the most widely prescribed drugs in the world, can cause gastrointestinal damage, including colitis. However, the prevalence of NSAID-induced colitis is unknown because the disease is often asymptomatic.

**Case presentation:**

We report the case of a 64-year-old female patient with a history of long-term NSAID use, who was hospitalized with septic shock caused by *Klebsiella pneumoniae* bacteremia. Computed tomography revealed multiple renal and splenic abscesses with diffuse colon wall thickening. A colonoscopy confirmed colitis with diffuse ulcers. NSAIDs were discontinued after this hospitalization. The abscesses improved after antibiotic treatment. A short course of balsalazide treatment was given under the suspicion of ulcerative colitis. Balsalazide was discontinued four months later due to a non-compatible clinical course. A follow-up colonoscopy two years later revealed a normal colon mucosa, and NSAID-induced colitis was diagnosed.

**Conclusion:**

This is the first reported case of combined bacterial splenic and renal abscesses without intestinal manifestations as the initial presentation of NSAID-induced colitis. In contrast to cases of *K. pneumoniae* bacteremia with primary liver abscesses in patients with diabetes mellitus in Taiwan, we presented the first case with abscesses caused by community-acquired *K. pneumoniae* in the kidneys and spleen without liver invasion*.* In conclusion, our case report alerts clinicians to the possibility that *K. pneumoniae* bacteremia combined with multiple abscesses can be associated with severe NSAID-induced colitis.

## Background

Non-steroidal anti-inflammatory drugs (NSAIDs), despite their well-known adverse effects on the gastrointestinal (GI) tract, are widely prescribed worldwide. NSAIDs damage different regions of the GI tract, including the distal small bowel and colon can be the target of NSAIDs. A study using fecal calprotectin (a non-degraded neutrophil cytosolic protein) to diagnose NSAID enteropathy found that the prevalence of NSAID enteropathy was around 44% [1]. Studies evaluating the colonic side effects of NSAIDs have suggested that NSAID-induced colitis is common; however, symptomatic NSAID-induced colitis is rare [2,3]. People taking NSAIDs are two to five times more likely to develop colonic inflammation than the general population. NSAIDs affect the large intestine by causing colonic ulceration and stricture formation. Approximately 10% of newly diagnosed colitis cases may be related to NSAID administration [4,5]. However, the prevalence of NSAID-induced colitis is unknown because the disease is often asymptomatic.

NSAID-induced colitis usually has nonspecific histological findings. The diagnosis of NSAID-induced colonic ulceration has been made based on a history of NSAID use and the exclusion of other causes [2]. The temporal relationship between NSAID use and symptoms that resolve after cessation confirm the diagnosis of NSAID-induced colitis.

Intra-abdominal abscesses as the initial presentation of NSAID-induced colitis has not been reported previously. Herein, we report the unusual case of a patient with renal and splenic abscesses but without intestinal symptom as the initial manifestations of NSAID-induced colitis.

## Case presentation

A 64-year-old Taiwanese woman presented with a history of well-controlled type 2 diabetes mellitus and hypertension. She had been self-treating regularly with NSAIDs from pharmacy (diclofenac, 50 mg three times daily) for more than two years because of chronic low back pain caused by intervertebral disc herniation. General malaise, poor appetite, intermittent postprandial nausea, urinary urgency and frequency were present one month before admission. She visited local clinics and received no specific diagnosis. Due to progressive shortness of breath and drowsiness for one week, she was sent to a medical center. She appeared acutely ill but afebrile, with a blood pressure of 74/34 mmHg, a pulse rate of 86/min, a respiratory rate of 22/min, and an oxygen saturation level of 93% in ambient air. Physical examination revealed no specific local findings. A blood assay revealed the following findings: leukocyte count, 44,400/μL with 85% neutrophils; hemoglobin, 12.3 g/dL; platelet count, 466, 000/μL; C-reactive protein, 235.76 mg/L; total bilirubin, 1.17 mg/dL; direct bilirubin, 0.17 mg/dL; aspartate aminotransferase, 38 IU/L; alanine aminotransferase, 21 IU/L; blood urea nitrogen, 96.77 mg/dL; creatinine, 7.8 mg/dL; sodium, 140 mmol/L; potassium, 8.6 mmol/L; and lactate, 20.3 mmol/L. The arterial blood gas analysis revealed severe metabolic acidosis (pH, 7.02; HCO_3_, 7.1 mmol/L). A chest radiograph revealed no pulmonary lesions. Urine analysis via catheterization revealed only mild hematuria.

**Figure 1:**
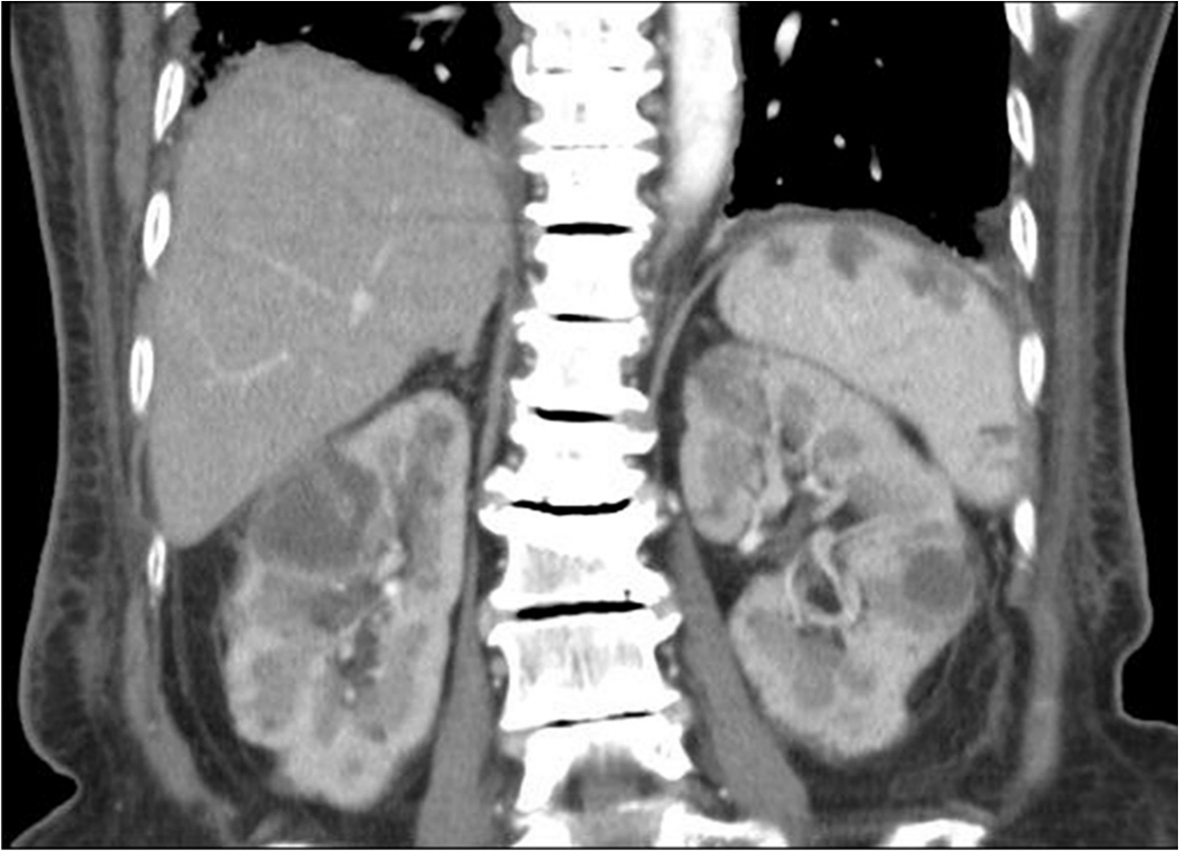
Computed tomography scans of the abdomen revealing several rim-enhancing hypodense lesions in bilateral kidneys and the spleen.

**Figure 2:**
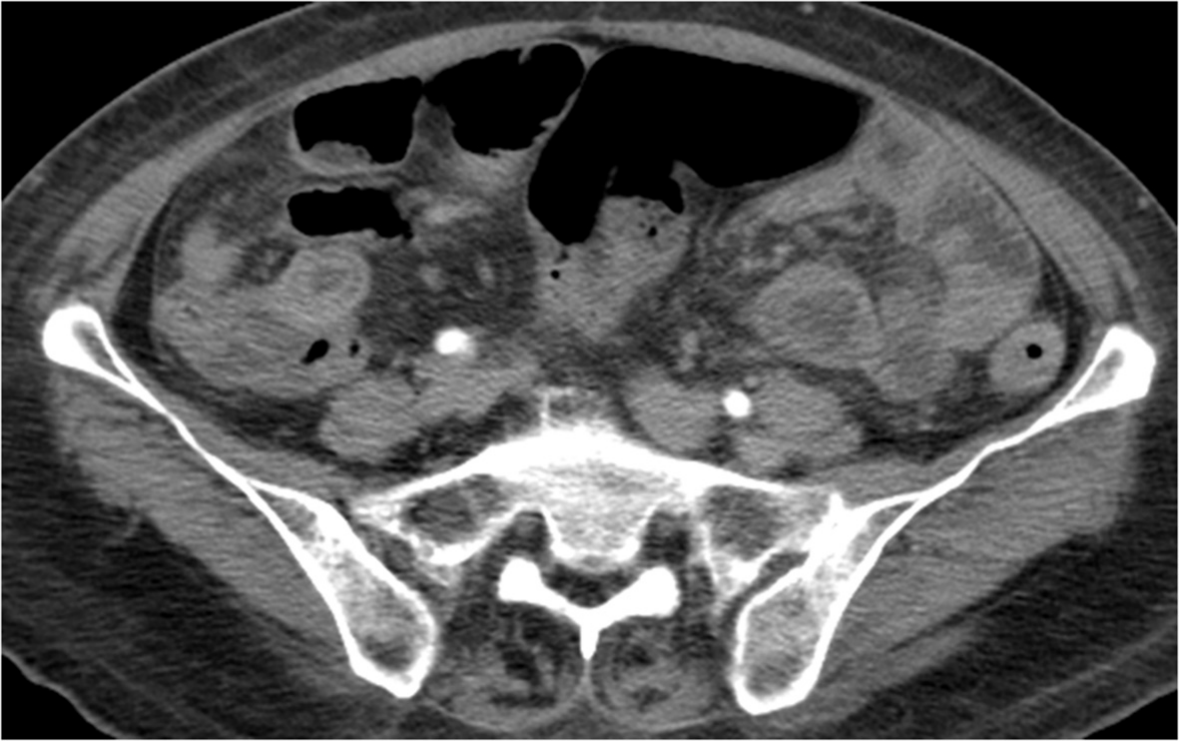
**Computed axial tomography scans of the abdomen showing marked thickening of the wall of the terminal ileum, cecum, ascending colon, descending colon, and rectum with perifocal fat stranding.** These finding are compatible with the findings of the colonoscopy.

**Figure 3:**
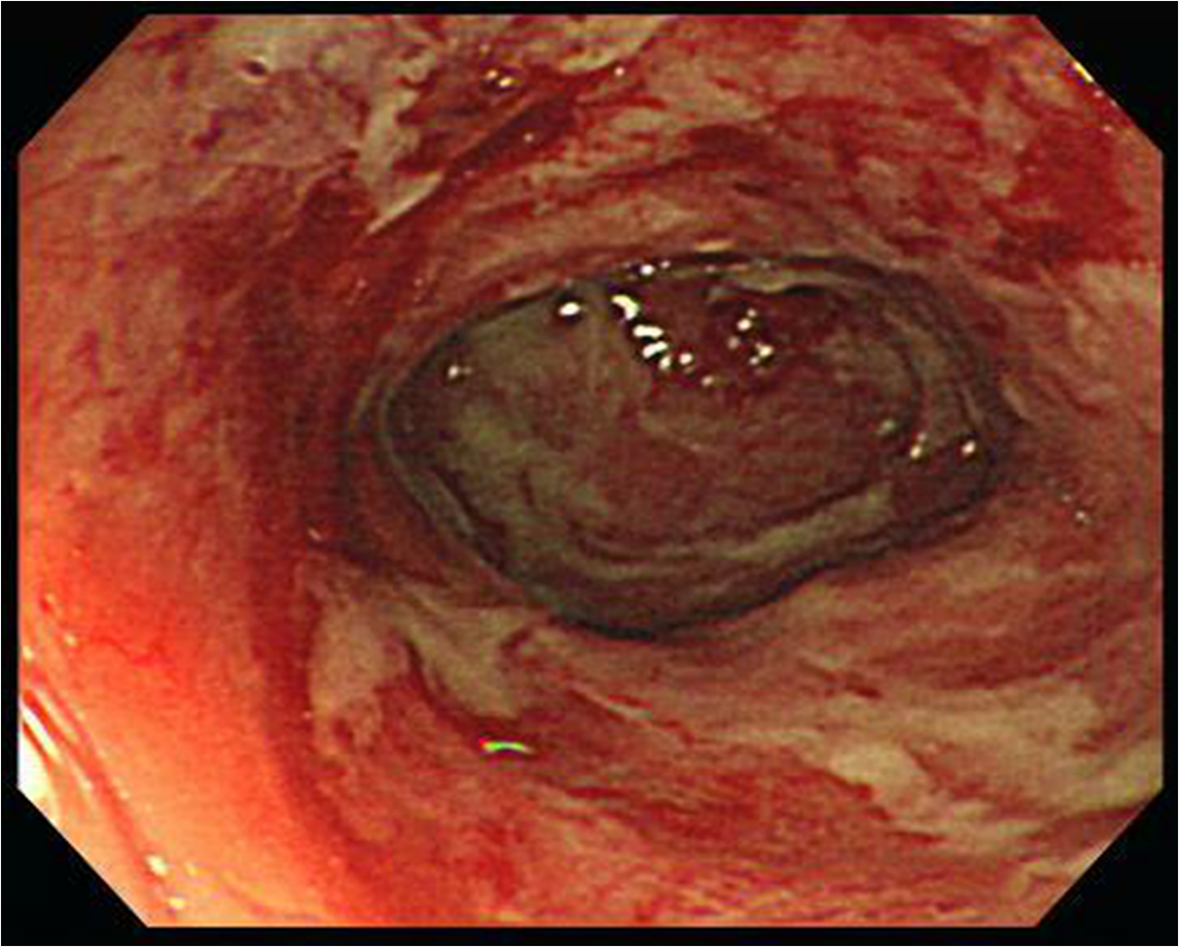
A colonoscopy revealed extensive segmental longitudinal ulceration of the mucosa extending over the rectum, sigmoid colon, and terminal ileum.

**Figure 4:**
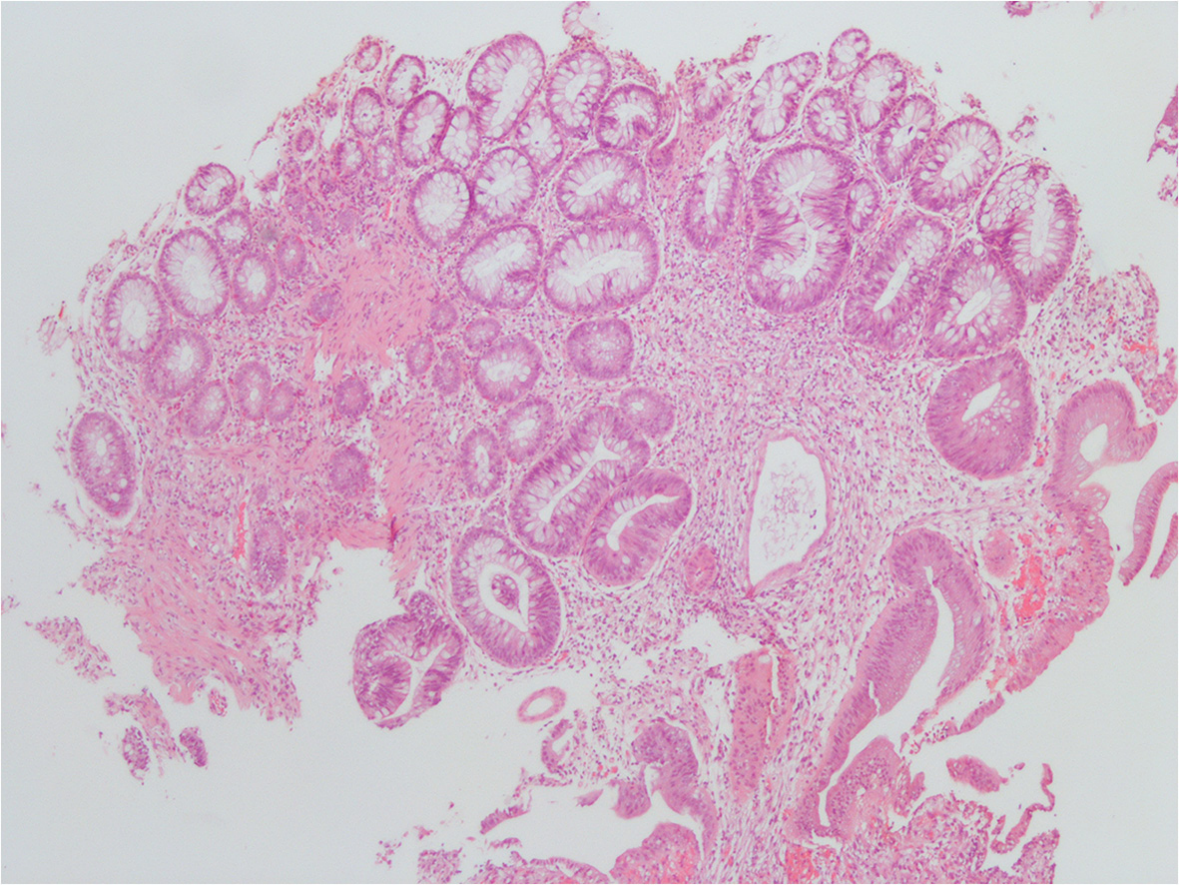
Photomicrograph of a sigmoid colon biopsy specimen displaying an ulcer, necrotic debris, and lymphocyte infiltration into the edematous lamina propria (hematoxylin and eosin, original magnification ×100).

She was admitted to the intensive care unit due to septic shock, acute respiratory failure, acute renal failure, and hyperkalemia. She received mechanical ventilation, intravenous vasopressors, continuous veno-venous hemofiltration, and cefmetazole (2 g/day) was prescribed empirically. All blood cultures yielded *K. pneumoniae*. The urine culture was sterile. Contrast-enhanced abdominal computed tomography (CT) revealed multiple abscesses in the spleen and bilateral kidneys, with the largest lesion (approximately 5.2 cm in diameter) located in the right kidney (Figure 1). In addition, CT also revealed segmental bowel wall edema in the submucosal layer over the terminal ileum, cecum, ascending colon, and descending colon with perifocal fat stranding. The hyperemic change was compatible with the inflammatory reaction in the bowel. The CT also revealed more intense wall enhancement at the terminal ileum (Figure 2). A colonoscopy revealed diffuse ulceration with exudation in the distal terminal ileum and segmental ulceration in the colon, with a reduction in the lumen caliber in the sigmoid colon (Figure 3). The pathological analysis of colon biopsy specimens revealed ulcers, necrotic debris, and the infiltration of lymphocytes into the edematous lamina propria (Figure 4). Three weeks later, a follow-up abdominal ultrasonography demonstrated partial resolution of the renal and splenic abscesses.

The patient was treated in the outpatient department with 500 mg of oral cephradine, four times daily; 500 mg of metronidazole, three times daily; and balsalazide (2.25 g daily) for two months. The follow-up abdominal CT scan revealed complete resolution of the abscesses two months later, and the colonoscopy revealed multiple segmental longitudinal ulceration with skip lesions in the rectum, sigmoid, and terminal ileum. A colon biopsy showed necrotizing inflammation with the infiltration of mixed inflammatory cells ,including neutrophils, histiocytes, and lymphocytes. Moreover, focal ulcerated colonic mucosa with inflammatory infiltration was found in the edematous, fibrous lamina propria. Some distorted glands were observed, but no crypt abscesses were noted. Balsalazide was discontinued after a course of four months. She had an uneventful course in the out-patient department, and a follow-up colonoscopy two years later revealed melanosis coli and internal hemorrhoid only, without ulcers or stenosis over the entire colon.

## Discussion

Our case is unique due to the presentation of severe colitis without an initial manifestation of intestinal symptoms. NSAID-induced colitis was diagnosed based on the history of long-term NSAID use after excluding other etiologies. To the best of our knowledge, this is the first reported case of NSAID-induced colitis with renal and splenic abscesses caused by community-acquired *K. pneumoniae* without liver involvement.

*K. pneumoniae*, a gram-negative encapsulated aerobic bacterium, is part of the normal flora of the human mouth and intestines. Human carrier rates in the community range from 5 to 38% in the stool and from 1 to 6% in the nasopharynx [6,7]. The infection rate is higher for individuals with impaired host defenses, such as individuals with diabetes mellitus and hepatobiliary tract disease [8,9]. *K. pneumoniae* has been reported to be the most common pathogen associated with pyogenic hepatic abscesses and splenic abscesses, especially in Taiwan and countries in Eastern and Southeast Asia [10-12], and this bacterium accounts for 10–16% of cases of splenic abscess and approximately 25% of cases of renal abscess [13,14].

Intra-abdominal abscesses have diverse symptoms. Fever and abdominal pain are the most frequent symptoms, but a few cases have vague symptoms, as in the case presented herein. Imaging analysis, including ultrasonography and computed tomography (CT), are necessary to diagnose intra-abdominal infections [15].

Splenic abscesses are rare, with a reported frequency of 0.14–0.7% in case reports [16]. Chang et al. analyzed 67 splenic abscess cases which occurred over a period of 19 years and found that *K. pneumoniae* was the most frequently encountered pathogen in blood cultures or abscess cultures, similar to observations from other Asian countries [11]. Ferraioli G. et al. found a different result; they observed that the most common pathogens were polymicrobial pathogens in pus cultures and *Staphylococcus* species in blood cultures [16].

The pathogenesis of intra-abdominal abscesses caused by *K. pneumoniae* remains unclear. One hypothesis for the pathogenesis of pyogenic intra-abdominal abscesses involves hematogenous bacterial seeding from the GI tract. An animal study demonstrated that *K. pneumoniae* strains with genetic regulatory networks for translocation have the ability to cross the intestinal barrier [17]. A careful search for the source of *K. pneumoniae* bacteremia other than primary bacteremia should be considered, and which should include colon lesions survey [18]. In our case, the finding of thickened colonic wall prompted us to consider the colon as the possible source of *K. pneumoniae* bacteremia.

Prostaglandins represent one of the most important components of mucosal defense in the colon and are involved in the maintenance of microcirculation and blood flow to modulate the mucosal immune system. NSAIDs inhibit colonic prostaglandin synthesis, leading to the development of colitis and the aggravation of preexisting intestinal diseases.

The ileocecal region is the most common site of NSAID-induced colonic injury. Pathological examination of colon specimens from patients with NSAID-induced colitis usually reveals sharply demarcated, semilunar or circumferential, superficial ulcerations with normal surrounding mucosa. There are no specific histopathological findings for NSAID-induced colitis except for the diaphragm-like stricture. Cellular infiltration into the lamina propria and epithelium may involve predominantly neutrophils or lymphocytes, depending on the chronicity of the lesion [19]. Symptomatic patients may have watery or bloody diarrhea, weight loss, fatigue, abdominal pain, anorexia, iron-deficiency anemia, and hypoalbuminemia.

There have been no studies to determine the risk of colitis related to different types of NSAIDs; however, many reports have stated that diclofenac, mefenemic acid, piroxicam, and ibuprofen could cause colorectal ulcers or colitis [4,20-22]. According to one case-controlled study, the long-term use of NSAIDs increased the risk of colonic mucosal lesions, suggesting that NSAIDs may contribute to the pathogenesis of colonic ulcers or colitis [23]. In addition, naproxen has been implicated in eosinophilic colitis [24], and diclofenac may be associated with pseudomembranous colitis [25].

In this case, the colonoscopy findings and pathological findings were not able to determine the etiology of the colitis. Due to evidence of a possible association between the use of NSAIDs and the exacerbation or relapse of inflammatory bowel diseases [26,27], NSAID-exacerbated inflammatory bowel disease was initially suspected. This patient was initially treated with balsalazide, but she became stable despite the discontinuation of balsalazide.

The discontinuation of NSAIDs is the cornerstone of the treatment of NSAID-induced colitis. Symptoms usually resolve within days to weeks of the withdrawal of NSAIDs, with the restoration of normal histology. Our patient received a colonoscopy two years later that revealed complete restoration of the colon mucosa; thus, this patient was diagnosed with NSAID-induced colitis.

## Conclusions

We present a unique case of NSAID-induced colitis without any GI symptoms at initial presentation, that was associated with septic shock caused by *K. pneumoniae* bacteremia with multiple renal and splenic abscesses. The colitis was found incidentally by CT and then identified as NSAID-induced colitis. This is also the first reported case of disseminated abscess formation caused by community-acquired *K. pneumoniae* in the kidneys and spleen without liver involvement in an NSAID-induced colitis patient.

This case is a reminder that hematogenous intra-abdominal abscesses caused by *K. pneumoniae* necessitate a thorough investigation of the GI tract. Assessing the presence of colitis should be considered even in the absence of preceding gastrointestinal symptoms, especially for those who have previously used NSAIDs.

## Consent

Written informed consent was obtained from the patient for publication of this case report and any accompanying images. A copy of the written consent is available for review by the Editor of this journal.

## Abbreviations

NSAIDs: Non-steroidal anti-inflammatory drugs; GI: Gastrointestinal; CT: Computed tomography.

## Competing interests

The authors declared that they have no competing interests.

## Authors’ contributions

HLH, WRL, and PLL prepared the manuscript. YHC and CHK provided laboratory support. WRL and CHK cared for the patient and provided advice on the clinical aspects of the case report. All authors read and approved the final version of the manuscript.

## Pre-publication history

The pre-publication history for this paper can be accessed here:

http://www.biomedcentral.com/1471-230X/13/139/prepub
